# Does one-anastomosis gastric bypass provide better outcomes than sleeve gastrectomy in patients with BMI greater than 50? A systematic review and meta-analysis

**DOI:** 10.1097/JS9.0000000000000203

**Published:** 2023-03-24

**Authors:** Maryam Barzin, Amir Ebadinejad, Ali Aminian, Alireza Khalaj, Faranak Ghazy, Fatemeh Koohi, Farhad Hosseinpanah, Amirhossein Ramezani Ahmadi, Majid Valizadeh, Behnaz Abiri

**Affiliations:** a Obesity Research Center, Research Institute for Endocrine Sciences, Shahid Beheshti University of Medical Sciences, Tehran, Iran; b Department of Surgery, Tehran Obesity Treatment Center, Faculty of Medicine, Shahed University, Tehran, Iran; c Endocrine and Metabolism Research Center, Isfahan University of Medical Sciences, Isfahan, Iran; d Department of General Surgery, Bariatric and Metabolic Institute, Cleveland Clinic, 9500 Euclid Avenue, M61, Cleveland, OH 44195, USA

**Keywords:** comorbidities, one-anastomosis gastric bypass, postoperative complications, sleeve gastrectomy, super obese, weight loss

## Abstract

In patients with BMI greater than 50, sleeve gastrectomy (SG) may not be adequate to treat obesity. To determine whether one-anastomosis gastric bypass (OAGB) can provide better outcomes compared with SG in patients with BMI greater than 50, a systematic review and meta-analysis was conducted, including a total of nine retrospective studies with a total of 2332 participants. There was a significant difference in the percentage of excess weight loss [weighted mean difference (WMD): 8.52; 95% CI: 5.81–11.22; *P*<0.001) and percentage of total weight loss (WMD: 6.65; 95% CI: 5.05–8.24; *P*<0.001). No significant differences were seen in operative time (WMD: 1.91; 95% CI: −11.24 to 15.07; *P*=0.77) and length of stay in hospital (WMD: −0.41; 95% CI: −1.18 to 0.37; *P*=0.30) between the two groups. There were no significant differences between OAGB with SG in Clavien–Dindo grades I–III [odds ratio (OR): 1.56; 95% CI: 0.80–3.05], or grade IV complications (OR: 0.72; 95% CI: 0.18–2.94). The meta-analysis on remission of type 2 diabetes indicated a comparable effect between SG and OAGB (OR: 0.77; 95% CI: 0.28–2.16). The OAGB group had a significantly higher rate of remission of hypertension compared with the SG group (OR: 1.63; 95% CI: 1.06–2.50). The findings of this meta-analysis suggest that the OAGB accomplished a higher percentage of total weight loss and percentage of excess weight loss at short-term and mid-term follow-up but, there was no major difference between the OAGB and SG operations in terms of perioperative outcomes, complications, and diabetes remission.

Highlights

- According to the result of the current systematic review and meta-analysis, the one-anastomosis gastric bypass (OAGB) accomplished a higher percentage of total weight loss and percentage of excess weight loss at short-term and mid-term follow-up.
- There was no major difference between the OAGB and sleeve gastrectomy operations in terms of perioperative outcomes, complications, and diabetes remission.
- Large sample and multicenter clinical trials are needed to compare the effectiveness and safety between OAGB and sleeve gastrectomy in patients with super obesity.

## Introduction

The worldwide prevalence of obesity has doubled since 1980, and if the current trends continue, it is estimated that by 2030 the absolute number of patients with obesity will be 1.12 billion^1^–^3^. A subset of patients with obesity that have a BMI above 50 is considered super obesity (SO)^4^. The growth rate of SO increased faster in the United States between 1986 and 2010 compared with the prevalence of BMI categories less than 50 kg/m^2^; also, individuals with SO are prone to more health problems such as type 2 diabetes mellitus (T2DM), hypertension (HTN), congestive heart failure, and chronic obstructive pulmonary disease^5^–^7^.

Bariatric surgery is considered a safe treatment for SO with durable weight loss and improvement of obesity-related comorbidities^8^–^10^. Sleeve gastrectomy (SG), Roux-en-Y gastric bypass (RYGB), and one-anastomosis gastric bypass (OAGB) are currently the most common bariatric operations, while there is no consensus among surgeons for SO^11^. Patients with SO need to lose a larger amount of their body weight. Therefore, more extensive bariatric operations may provide better clinical outcomes for these patients^10^. On the other hand, bariatric surgery in SO patients is usually technically challenging due to anesthesia risk, abundant visceral fat, larger liver size, and the strong force required to work with the instruments^12^,^13^. A recent meta-analysis demonstrated that RYGB was superior to SG in resolving comorbidities and weight loss for SO at 12 months; however, during a longer follow-up time of around 24 months, RYGB was comparable to SG^9^. But, another meta-analysis concluded that RYGB may be superior to SG in terms of long-term outcomes^14^.

OAGB is a simple gastric bypass procedure associated with acceptable operation risks and weight loss results in morbid obesity patients^15^. There are several studies among SO patients, particularly comparing the results of SG and OAGB, and no meta-analysis has been performed yet. This systematic review and meta-analysis aimed to compare the safety and effectiveness of OAGB and SG in patients with SO. We hypothesized that OAGB could provide better clinical outcomes compared with SG in patients with BMI greater than 50.

## Methods

This systematic review was performed according to the Preferred Reporting Items for Systematic reviews and Meta-Analyses (PRISMA) statement^16^ and Assessing the Methodological quality of Systematic Reviews (AMSTAR) guideline^17^. The review protocol was registered at the PROSPERO (registration number CRD42021286864). The prospective registration identifies comorbidity changes, operative time, and length of stay as important outcomes.

### Search strategy

We systematically reviewed the English-language literature published until October 2021 by searching relevant keywords in PubMed, Scopus, Cochrane Clinical Trials Registry, and Web of Science electronic databases. The following terms were used in medical subject headings and free-text searches: (‘mini gastric bypass’ OR single anastomosis gastric bypass OR omega loop gastric bypass OR one anastomosis gastric bypass), (‘super obes*’ OR ‘BMI 50’ OR ‘body mass index 50’). We attempted to identify additional studies by searching the reference lists of relevant articles. If necessary, we contacted the authors to acquire further information.

### Study selection

All cohort studies and case series were included in the analysis. Case reports, review articles, abstracts, and systematic reviews or papers published in non-English languages and animal studies were excluded from the final analysis. Studies were included if they met the following criteria: (1) studies that examined the population of obese adults (BMI ≥50 kg/m^2^); (2) studies that have compared OAGB and SG; (3) studies that have shown results in the weight loss or improvement in comorbidities or postoperative complications.

### Data extraction

After the search was conducted and selecting the appropriate literature, two researchers (A.E. and F.G.) independently extracted data from the included literature. If there were disagreements about included studies, a third reviewer (B.A.) was available to arbitrate. A standardized data extraction form was developed. The following information was extracted from each article: first author, publication year, number of patients, age of participants, time of follow-up, preoperative BMI, percentage of excess weight loss (%EWL) and total weight loss (%TWL), postoperative resolution of T2DM and HTN, complications (according to Clavien–Dindo classification)^18^, number of deaths, and operative time.

### Quality assessment

To evaluate the quality of the observational studies, we employed the nine-point Newcastle–Ottawa Scale^19^, which evaluates three fundamental aspects of methodology: study participant selection (0–4 points), confounder adjustment (0–2), and outcome indicator determination (0–3). A study with a score of 7–9 points was defined as high quality.

### Statistics

All statistical analyses were performed using data analysis and statistical software STATA version 12.0. Mean differences in perioperative outcomes (operative time and length of hospital stay), %EWL, and %TWL after OAGB and SG were pooled using the random-effect model. The incidence of overall complication and remission of diabetes mellitus and HTN were investigated. The measure of the effect of interest was the odds ratio (OR) with a 95% CI. We used the Mantel–Haenszel method for the calculation of the OR. Studies with data on rates of perioperative complications according to the Clavien–Dindo classification were included in the analysis.

Missing mean and SDs were calculated from other statistics if needed, such as values of mean change from baseline or baseline SDs. In addition, when data were reported as median±interquartile range, assuming a normal distribution of data, the median was considered as mean, and SD was calculated as interquartile range/1.35. The *I* ^2^ statistics were used to test statistical heterogeneity among studies. A study with a heterogeneous source, due to differences in methodology or other reasons, was excluded from the analysis to determine the robustness of the observed outcomes to the assumptions made in performing the analysis. Publication bias was assessed by the Egger weighted regression method; a *P* value of less than 0.1 was considered representative of statistically significant publication bias.

## Results

### Search results and baseline characteristics

**Figure 1:**
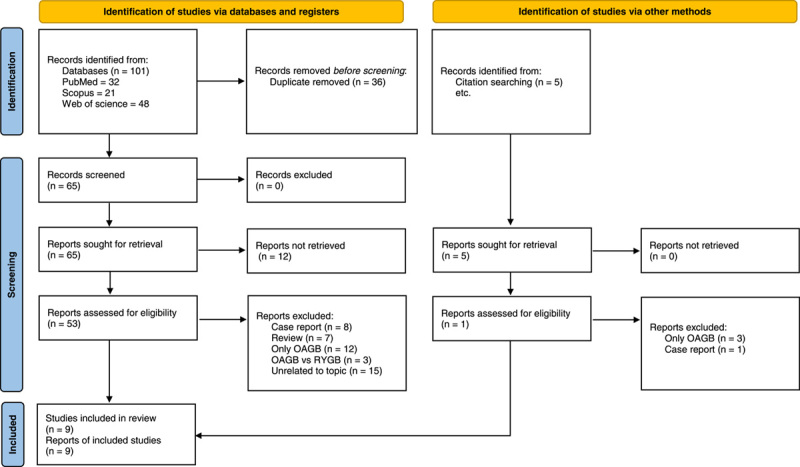
PRISMA flow diagram for the selection process of the studies. OAGB, one-anastomosis gastric bypass; RYGB, Roux-en-Y gastric bypass.

A total of 101 articles were identified in the search, the titles and abstracts of the articles were screened, and only 53 were deemed potentially eligible. After a review of full-text articles and abstracts, nine retrospective studies were eligible for inclusion which were published from 2016 to 2021. The selection process is shown in Figure 1. The nine studies included 1989 participants (OAGB: 893, SG: 1989).

The mean age in the OAGB and SG groups were 41.2±3.8 and 41.9±4.8 years, respectively. The preoperative BMI of participants in the OAGB and SG groups were 57.1±7.1 and 57.7±7.4 kg/m^2^, respectively. The minimum and maximum sample size of patients analyzed ranged from 33 to 557, and the time to follow-up ranged from 6 to 60 months. The characteristics of the nine included studies^20^–^28^ are shown in Table 1. The quality score of each included study is presented in Table 2.

**Table 1:** General characteristics of the included studies in the systematic review and meta-analysis

References	Study design	Number of patients (OAGB/SG)	Age (mean±SD) (OAGB vs. SG)	Time of follow-up (months)	Preoperative BMI (OAGB vs. SG)	%EWL/%TWL (OAGB vs. SG)	Postoperative DM/HTN resolution (OAGB vs. SG)	Complications^a^ (OAGB vs. SG)	Mortality^b^ (OAGB vs. SG)	Operation time^c^ (min) (OAGB vs. SG)
Soong *et al*.^20^	Retrospective	436 (246/190)	31.9±9.7 vs. 33.0±10.0	12 and 60	56.2±5.8 vs. 55.9±5.5	64.5/38.7 vs. 59.5/35.7 and 68.0/40.7 vs. 60.3/35.1	NM/NM vs. NM/NM and 100%/34.8% vs. 100%/20.4%	21 vs. 6	0 vs. 0	140.1 vs. 133.7
Tasdighi *et al*.^21^	Retrospective	557 (209/348)	40.4±10.9 vs. 39.2±12.6	12, 24, and 36	54.9±4.6 vs. 54.3±3.9	66.7/36.6 vs. 61.7/33.2 and 70.9/36.8 vs. 62.1/33.6 and 72.3/39.5 vs. 59.3/32.1	NM	32 vs. 15	0 vs. 0	121.9 vs. 109.7
Rajan *et al*.^22^	Retrospective	30 (3/27)	42.3±10.7 vs. 39.2±11.2	12	52.8±1.5 vs. 60.9±9.3	NM	NM	0 vs. 1	0 vs. 0	116.2 vs. 103.5
Schmitz *et al*.^23^	Retrospective	243 (150/93)	39.11±0.9 vs. 41.57±1.07	12	64.14±0.3 vs. 66.91±0.6	NM	51%/98% vs. 42%/69%	4 vs. 11	0 vs. 0	81.36 vs. 92.08
Abouelela *et al*.^24^	Retrospective	50 (25/25)	44.87±10.34 vs. 45.11±9.09	6 and 12	65.12±5.89 vs. 67.12±3.95	59.11/NM vs. 79.76/NM and 58.11/NM vs. 76.11/NM	NM/NM vs. NM/NM and 80%/60% and 76%/64%	2 vs. 2	0 vs. 0	82.89 vs. 62.11
Singla *et al*.^25^	Retrospective	75 (25/50)	39.56±9.77 vs. 40.95±9.77	12	53.76±3.28 vs. 54.18±4.06	74.57/39.90 vs. 56.20/30.09	77.8%/78.6% vs. 85.8%/66.7%	0 vs. 2	0 vs. 0	NM
Bhandari *et al*.^26^	Retrospective	351 (124/227)	42.41±11.0 vs. 45.85±12.2	24 and 36	54.23±3.69 vs. 56.39±6.11	80.21/43.00 vs. 74.24/36.90 and 78.59/40.34 vs. 62.38/30.73	NM	8 vs. 3	0 vs. 1	55.96 vs. 36.12
Plamper *et al*.^27^	Retrospective	287 (169/118)	43.2±11.1 vs. 43.4±11.2	12	54.1±6.6 vs. 54.6±10.3	66.2/NM vs. 57.3/NM	NM	5 vs. 11	0 vs. 1	81.7 vs. 112.1
Madhok *et al*.^28^	Retrospective	75 (19/56)	45.0±25.0 vs. 51±30.0	6, 12, and 24	67.0±60.0 vs. 65.0±60.0	44.0/31.0 vs. 36.0/25.0 and 58.0/39.0 vs. 45.0/29.0 and 66.0/44.0 vs. 38.0/26.0	After 24 mo: 66.7%/12.5% vs. 53%/14.3%	2 vs. 8	0 vs. 0	92 vs. 75

^a^ Surgical complication according to Clavien–Dindo classification (grades Ι–ΙV). Data were reported as the total number of patients with grades Ι–ΙV complications.

^b^ Mortality or Clavien–Dindo grade V. Data were reported as the total number of mortality occurrence or Clavien–Dindo grade V.

^c^ Operation time was shown as mean.

DM, diabetes mellitus; EWL%, percentage of excess weight loss; HTN, hypertension; NM, not mentioned; OAGB, one-anastomosis gastric bypass; post-op, postoperation; pre-op, preoperation; SG, sleeve gastrectomy; TWL%, percentage of total weight loss.

**Table 2:** Quality assessment of the included studies

			Newcastle–Ottawa scale		
References	Study type	Number of patients	Selection	Comparability	Outcome
Soong *et al*.^20^	Retrospective	436	***	*	*
Tasdighi *et al*.^21^	Retrospective	557	***	*	***
Rajan *et al*.^22^	Retrospective	30	**	*	*
Schmitz *et al*.^23^	Retrospective	243	*	*	*
Abouelela *et al*.^24^	Retrospective	50	***	*	**
Singla *et al*.^25^	Retrospective	75	**	*	**
Bhandari *et al*.^26^	Retrospective	351	**	*	**
Plamper *et al*.^27^	Retrospective	287	*	*	*
Madhok *et al*.^28^	Retrospective	75	***	*	*

### Percentage of excess weight loss and total weight loss at less than and more than 12 months of follow-up

**Figure 2:**
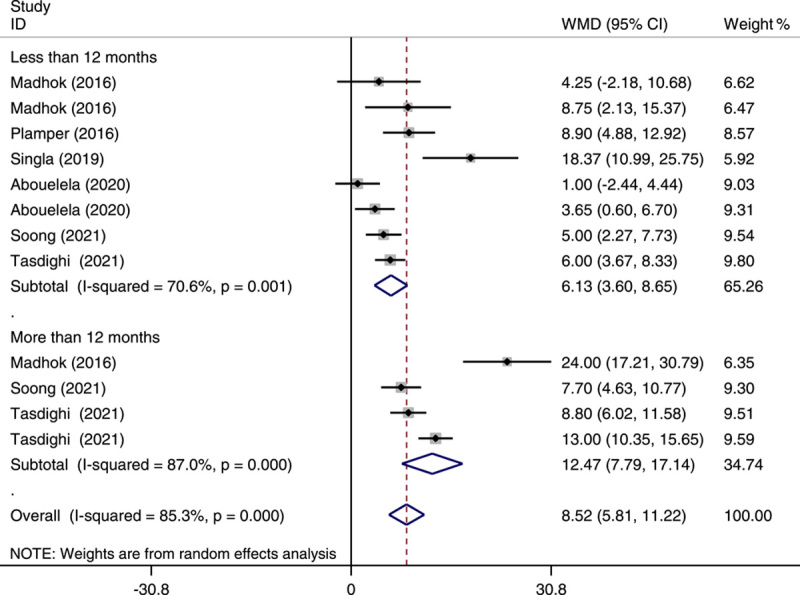
Forest plot of pooled weighted mean difference (WMD) and its 95% CI for studies comparing one-anastomosis gastric bypass with sleeve gastrectomy in the percentage of excess weight loss by postoperative follow-up period. The right side of the vertical line refers to more percentage of excess weight loss for one-anastomosis gastric bypass than sleeve gastrectomy and vice versa.

**Figure 3:**
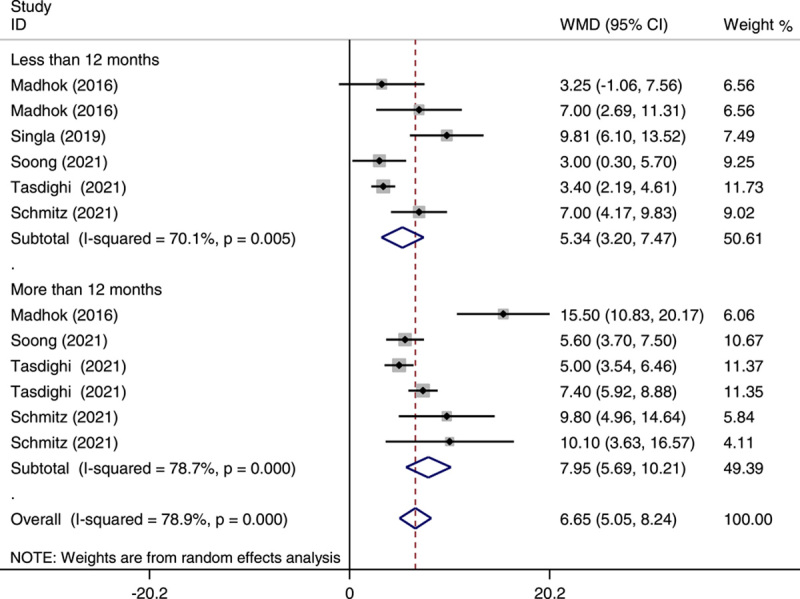
Forest plot of pooled weighted mean difference (WMD) and its 95% CI for studies comparing one-anastomosis gastric bypass with sleeve gastrectomy in the percentage of total weight loss by postoperative follow-up period. The right side of the vertical line refers to more percentage of total weight loss for one-anastomosis gastric bypass than sleeve gastrectomy and vice versa.

As demonstrated in Figure 2, of the studies included in the meta-analysis OAGB showed a higher %EWL compared with SG at less than 12 months (WMD: 6.13; 95% CI: 3.60–8.65; *P*<0.001) and longer than 12 months of follow-up (WMD: 12.47; 95% CI: 7.79–17.14; *P*<0.001), as well as in overall (WMD: 8.52; 0.95% CI: 5.81–11.22; *P*<0.001). Additionally, our meta-analysis showed a significant %TWL in OAGB compared with SG at less than 12 months (WMD: 5.34; 95% CI: 3.2–7.47; *P*<0.001) and longer than 12 months of follow-up (WMD: 7.95; 95% CI: 5.69–10.21; *P*<0.001), as well as in overall (WMD: 6.65; 0.95% CI: 5.05–8.24; *P*<0.001) (Fig. 3). There was significant heterogeneity in the pooled analysis in %EWL (*I* ^2^=85.3%) and %TWL (*I* ^2^=78.9%).

### Perioperative results and complications

**Figure 4:**
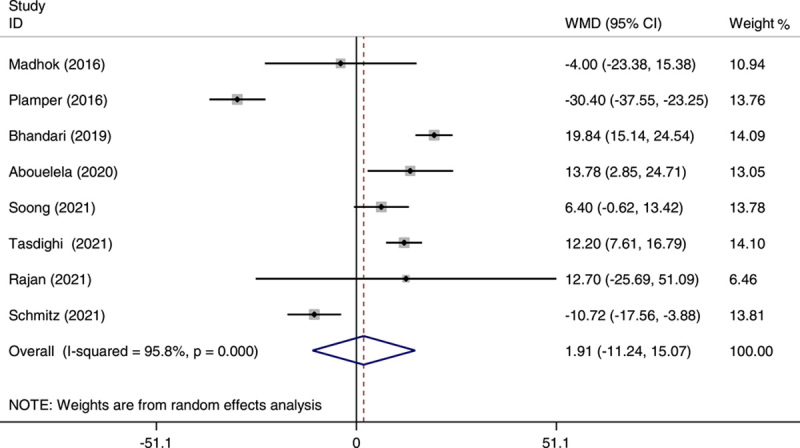
Forest plot of pooled weighted mean difference (WMD) and its 95% CI for studies comparing one-anastomosis gastric bypass (OAGB) with sleeve gastrectomy (SG) in operation time. The right side of the vertical line refers to a longer operation time for OAGB than SG and the left side refers to a shorter operation time for OAGB than SG.

**Figure 5:**
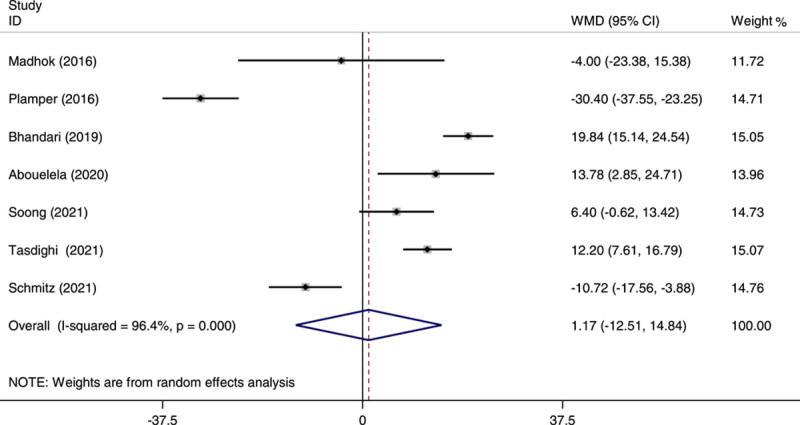
Forest plot of pooled standardized mean difference (SMD) and its 95% CI for studies comparing one-anastomosis gastric bypass (OAGB) with sleeve gastrectomy (SG) in operation time. The right side of the vertical line refers to a longer operation time for OAGB than SG and the left side refers to a shorter operation time for OAGB than SG (sensitivity analysis after removing Rajan’s study).

Of all the studies included in our analysis, those contained data on operative time, showing shorter operating times for SG than OAGB, except two studies that reported shorter operative time for OAGB^23^,^27^. However, the overall meta-analysis on the operative time indicated no significant difference between OAGB and with SG group (WMD: 1.91; 95% CI: −11.24 to 15.07; *P*=0.0.77) (Fig. 4). Even after excluding Rajan’s study^22^ (WMD: 1.17; 95% CI: −12.51 to 14.84; *P*=0.86), the results remained nonsignificant (Fig. 5).

**Figure 6:**
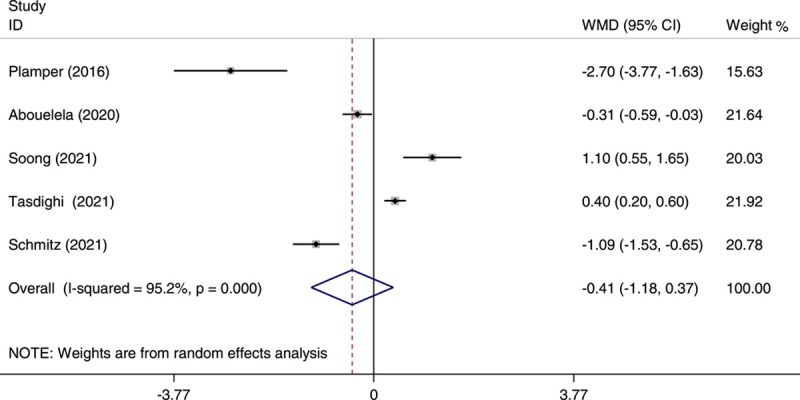
Forest plot of weighted mean difference (WMD) and its 95% CI for studies comparing one-anastomosis gastric bypass (OAGB) with sleeve gastrectomy (SG) in the length of stay in hospital. The right side of the vertical line refers to a longer length of stay in the hospital for OAGB than SG and the left side refers to a shorter length of stay in the hospital for OAGB than SG.

Of all the studies included in the meta-analysis, five studies reported data on the length of hospital stay^20^,^21^,^23^,^24^,^27^. The overall analysis showed a nonsignificant difference between the two groups (WMD: −0.41; 95% CI: −1.18 to 0.37; *P*=0.30) (Fig. 6).

**Figure 7:**
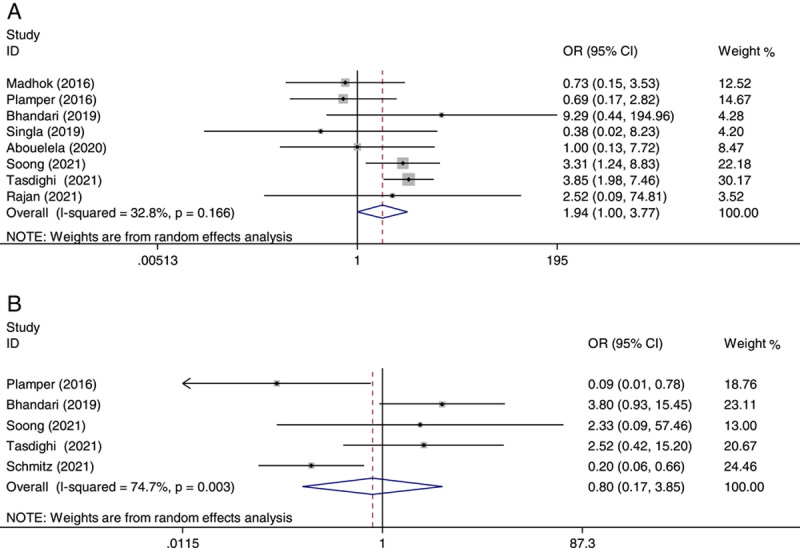
Forest plot of odds ratio (OR) and its 95% CI for studies comparing one-anastomosis gastric bypass with sleeve gastrectomy in Clavien–Dindo classification grades I–III (panel A) and Clavien–Dindo classification grade IV (panel B). The right side of the vertical line refers to more Clavien–Dindo classification grades for one-anastomosis gastric bypass compared to sleeve gastrectomy and vice versa.

As demonstrated in Figure 7, there were no significant differences between OAGB with SG in Clavien–Dindo classification grades I, II, and III complications (OR: 1.56, 95% CI: 0.80–3.05) (panel A)^20^–^22^,^24^–^28^, and Clavien–Dindo classification grade IV adverse events (OR: 0.72, 95% CI: 0.18–2.94) (panel B)^20^,^21^,^23^,^26^,^27^.

Furthermore, we excluded the study conducted by Schemitz *et al*.^23^ in panel B (OR: 1.05, 95% CI: 0.23–4.86) and also, Palmper’s study^27^ in panel B (OR: 1.08, 95% CI: 0.25–4.72), at a time and analyzed their impacts on the main summary estimate. In this sensitivity analysis, neither the heterogeneity nor the outcomes were significantly influenced by a single study.

The heterogeneity in the analysis for operation time was significant (*I* ^2^=95.8%), even after removing Rajan’s study^22^ (*I* ^2^=96.4%). The heterogeneity in the analysis for the length of hospital stay was significant (*I* ^2^=95.2%), but after removing Schmitz’s study^23^, it was not significant (*I* ^2^=6.2%). In regard to the Clavien–Dindo classification grades I–III and grade IV, the heterogeneity was significant for both classifications (*I* ^2^=52.2% and *I* ^2^=68.8%, respectively). The heterogeneity remained significant after removing the studies by Schmitz (*I* ^2^=59.9%) and Palmper (*I* ^2^=67.1%) in panel B. There was significant heterogeneity in the pooled analysis of early complications (*I* ^2^=71.2%).

### Remission of obesity-related comorbidities

**Figure 8:**
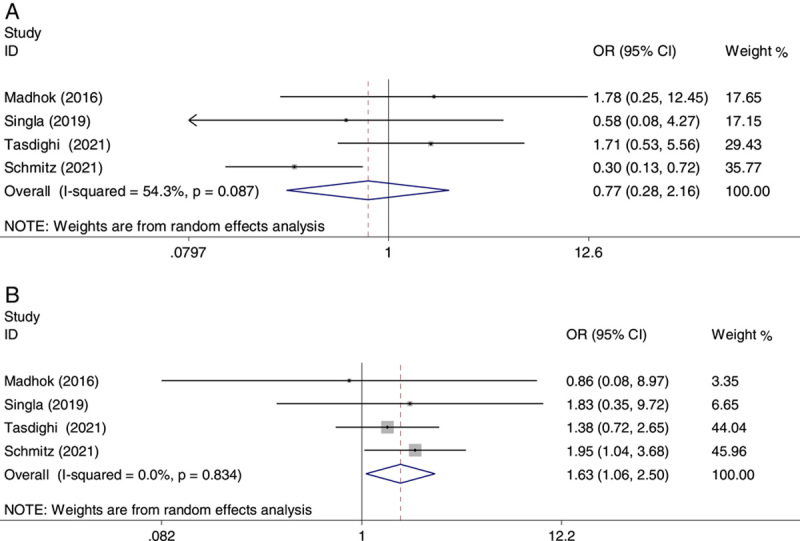
Forest plot of odds ratio (OR) and its 95% CI for studies comparing one-anastomosis gastric bypass with sleeve gastrectomy in obesity-related comorbidities resolution; panel A, diabetes; panel B, hypertension. The right side of the vertical line refers to more comorbidities resolution for one-anastomosis gastric bypass compared to sleeve gastrectomy and vice versa.

A total of four studies presented data on comorbidity remission at 12 months, including T2DM and HTN^21^,^23^,^25^,^28^. Based on the analysis, as a whole, the OAGB group had a significant and higher remission of HTN compared with the SG group (OR: 1.63, 95% CI: 1.06–2.50) (Fig. 8, panel B). After OAGB, 50.2% of patients did not have HTN at 1-year follow-up compared with 38.2% of SG patients.

The meta-analysis on T2DM remission indicated a comparable effect between OAGB and SG (OR: 0.77, 95% CI: 0.28–2.16) (Fig. 8, panel A). In this study, the meta-analysis of additional comorbidities was not possible because of the few data. There was no significant heterogeneity in the pooled analysis in the remission of HTN (*I* ^2^=0.0%) but, the heterogeneity in the analysis for T2DM was significant (*I* ^2^=54.3%).

### Publication bias

By the Egger weighted regression method, no publication bias was found in the analysis for perioperative results (operation time, *P*=0.73; length of hospital stay, *P*=0.24; Clavien–Dindo classification grades I, II, and III, *P*=0.34; Clavien–Dindo classification grade IV, *P*=0.76), remission of comorbidities (HTN, *P*=0.56, and T2DM, *P*=0.74), except for the overall %EWL (*P*=0.001).

## Discussion

This systematic review and meta-analysis included nine studies and compared the two weight loss surgical methods, SG and OAGB, on patients with SO. Favorable weight loss in the short and medium term of follow-up was seen after OAGB compared to SG. The remission of T2DM was similar between SG and OAGB, whereas remission of HTN was significantly better after OAGB. There was no difference between the two groups regarding the safety of the two operations, including the operative time, length of stay in the hospital, and surgical complications.

Bariatric surgery is the mainstay of treatment for SO^8^,^9^. SG and RYGB are currently the most popular bariatric surgeries^11^. OAGB has received much attention in recent years due to its simplicity of technique and acceptable results^29^. The body of evidence suggested that in people with obesity, OAGB resulted in better weight loss and improvement in comorbidities than SG^30^. A meta-analysis on patients with SO indicated that RYGB was superior to SG in inducing weight loss and control of T2DM and dyslipidemia at 12 months after surgery; however, weight loss after RYGB and SG were comparable in longer follow-up time^9^. But, another meta-analysis concluded that RYGB may be superior to SG in terms of long-term outcomes^14^. Parmar *et al*.^8^, in a systematic review, reported that OAGB in patients with SO in terms of safety and efficacy is an acceptable procedure.

Our meta-analysis found considerable weight loss following both procedures, although OAGB accomplished a higher TWL% and EWL% at 12 months and mid-term follow-up. Only Soong *et al*.^20^ examined the results of SG and OAGB after 5 years, which showed that weight loss results favored OAGB surgery. Similarly, in previous studies of patients with morbid obesity, OAGB had a greater EWL% of 1–5 years compared with SG^30^. The biliopancreatic limb length (BPL) in OAGB is one factor that determines the outcome of the surgery^31^. Patients underwent OAGB in two studies with a BPL of less than 200 cm, three studies with a BPL of 200 cm, two studies with a BPL of 250 cm, and one with a tailored BPL, which could be one of the causes of heterogeneity in this study. Only Tasdighi *et al*.^21^ investigated the effect of BPL length on outcomes which reported no difference between the procedures.

HTN was improved by 50.2% after OAGB and 38.2% after SG. There was no difference between the two operations in the remission of T2DM, which would be a surprising finding and should be considered with some caution due to the small number of diabetic patients and the lack of exact definitions for the remission of comorbidities in some studies. Previous studies in people with a BMI greater than 35 kg/m^2^ have shown that T2DM improved better in OAGB than in SG due to more pronounced weight loss and gastrointestinal hormone alteration in OAGB^30^,^32^. In our previous studies in patients with BMI greater than 35 study, the remission rate of HTN was significantly higher in the OAGB than in the SG^30^. Improvement in HTN days after surgery and before weight loss has been reported due to modifications in gastrointestinal hormones^33^. In this respect, glucagon-like peptide 1 and peptide YY increase after both procedures but more intensely after gastric bypass^33^. It should be noted that despite the low heterogeneity, the findings related to comorbidities are not conclusive due to the large CIs.

Bariatric surgery is still challenging for patients with SO. Anesthesia risk due to comorbidities and physiological abnormalities can be more significant in these patients^13^. Therefore, operative time is critical in choosing the type of surgery for this population. Studies have used different definitions to estimate the operative time, which is the leading cause of heterogenicity; In some publications, only the time of surgery and in others, the time of anesthesia with surgery have been reported. In OAGB, operative time ranged from 55.9 to 140.1 minutes, and in SG, from 36.1 to 133.7 minutes. The current pooled analysis showed no significant difference between the two operations. Similarly, a previous meta-analysis of patients with morbid obesity showed that OAGB and SG groups had comparable operative times and hospital stays^30^.

Likewise, complications after surgery are not reported with a uniform methodology in articles; we placed all the reported complications of studies in one of the five categories of Clavien–Dindo classification set up for bariatric operation and compared SG and OAGB^19^. Grade V complications or mortality in SG were seen in two patients but in none of the patients in the OAGB group. Regarding grade IV complications or complications that require therapeutic intervention, there was no significant difference between the two OAGB and SG groups. In addition, there was no difference between the two surgical groups in terms of complications that did not require intervention (grades I–III).

To the best of our knowledge, the present systematic review and meta-analysis is the first one that compared OAGB and SG for patients with SO. Our study has several notable limitations. First, there are no RCTs, and all the studies were cohort retrospective studies with inherent selection bias. Second, a small sample size, and short and variable follow-up time may influence the stability of the result. Third, studies do not use the same methods to define and express the surgery results, which leads to misinterpretation of the outcomes. Fourth, heterogeneity between studies was high in our meta-analysis, which may be explained by different baseline characteristics of included studies and different surgical techniques, particularly in the length of the BPL limb after OAGB and oversewing of staple line after SG. In addition, outcomes were relatively short-term and early weight loss reductions with OAGB may lead to improved long-term metabolic/comorbidity resolution compared to SG. Finally, it can be stated that the learning curve for OAGB is greater than SG which may contribute to the findings.

## Conclusion

As a whole, OAGB and SG were identified as effective procedures in weight loss and improvement of comorbidities, along with acceptable complication rates in patients with SO. The OAGB accomplished a higher TWL% and EWL% at short-term and mid-term follow-up. There was no major difference between the two operations in terms of perioperative outcomes and diabetes remission. However, the results of this meta-analysis should be interpreted cautiously according to the mentioned limitations, and the results are not conclusive. Large sample and multicenter clinical trials are needed to compare the effectiveness and safety between OAGB and SG in patients with SO.

## Ethical approval

Not applicable.

## Informed consent statement

Informed consent does not apply.

## Sources of funding

None.

## Conflicts of interest disclosure

No conflict of interest.

## Author contribution

A.E., F.H., B.A., M.V., F.K., and A.R.A. contributed to the design of the study, conducted the searches, screening, quality appraisal, data extraction, analysis, synthesis, and drafted and edited the manuscript. A.E. and F.G. conducted the data extraction. M.B. and A.K. contributed to the design of the study, supported screening, and revised the manuscript. M.V. and F.H. advised and revised the manuscript. B.A. and M.V. have primary responsibility for the final content. All authors have read and approved the final version of the manuscript.

## Research registration unique identifying number (UIN)

1. Name of the registry: PROSPERO.

2.Unique identifying number or registration ID: CRD42021286864.

3.Hyperlink to your specific registration (must be publicly accessible and will be checked): https://www.crd.york.ac.uk/prospero/display_record.php?ID=CRD42021286864

## Guarantor

Majid Valizadeh and Behnaz Abiri.

## Provenance and peer review

Not commissioned, externally peer-reviewed.

## Data statement

The data in this review is not sensitive in nature and is accessible in the public domain. The data is therefore available and not of a confidential nature.
